# Incidence and severity of SARS-CoV-2 infection and vaccine BNT162 side effects in children and adolescents with Noonan Syndrome: a national multicentric study

**DOI:** 10.3389/fped.2025.1658340

**Published:** 2026-01-12

**Authors:** Sarah Dal Ben, Federica Tamburrino, Francesca Bonomo, Emanuela Scarano, Eleonora Orlandini, Gabriella Pozzobon, Claudia Giavoli, Chiara Corezzola, Maria Felicia Faienza, Giuseppa Patti, Donatella Capalbo, Mariacarolina Salerno, Roberto Franceschi, Silvia Longhi, Malgorzata Gabriela Wasniewska, Domenico Corica, Thomas Zoller, Michele Piazza, Mohamad Maghnie, Franco Antoniazzi, Rossella Gaudino

**Affiliations:** 1 Department of Surgical Sciences, Dentistry, Gynecology and Pediatrics, University of Verona, Verona, Italy; 2 Pediatric Unit, IRCCS Azienda Ospedaliero-Universitaria di Bologna, Bologna, Italy; 3 Department of Pediatrics, Endocrine Unit, IRCCS San Raffaele Scientific Institute, Milan, Italy; 4 Endocrinology Unit, Fondazione IRCCS Ca’ Granda Ospedale Maggiore Policlinico, Milan, Italy; 5 Department of Clinical Sciences and Community Health, University of Milan, Milan, Italy; 6 Pediatric Unit, Department of Precision and Regenerative Medicine and Ionian Area, University of Bari “Aldo Moro”, Bari, Italy; 7 Department of Neuroscience, Rehabilitation, Ophthalmology, Genetics, Maternal and Child Health, University of Genoa, Genoa, Italy; 8 Department of Pediatrics, Endocrine Unit, IRCCS Istituto Giannina Gaslini, Genova, Italy; 9 Unit of Pediatric Endocrinology, Department of Medical and Translational Sciences, University of Naples Federico II, Naples, Italy; 10 Unit of Pediatrics, Department of Mother and Child, University Hospital of Naples Federico II, Naples, Italy; 11 Centre for Medical Sciences—CISMed, University of Trento, Trento, Italy; 12 Department of Pediatrics, Hospital of Bolzano (SABES-ASDAA), Teaching Hospital of Paracelsus Medical University, Bolzano, Italy; 13 Department of Human Pathology of Adulthood and Childhood, University of Messina, Messina, Italy; 14 Unit of Pediatrics, Department of Mother and Child, University Hospital, Messina, Italy; 15 Unit of Pediatrics, Department of Mother and Child, University Hospital of Verona, Verona, Italy

**Keywords:** BNT162, COVID-19, Noonan syndrome, pandemic, SARS-CoV-2, vaccine

## Abstract

**Background:**

Pre-existing medical conditions are known to increase the risk of severe coronavirus disease 2019 (COVID-19), even in pediatric populations. This study aimed to evaluate the symptoms and severity of COVID-19, as well as the side effects of the BNT162 vaccine, in children and adolescents with Noonan Syndrome (*N*S) compared to healthy controls.

**Methods:**

A retrospective and prospective multicenter study was conducted across Italy. Clinical characteristics, course, and duration of SARS-CoV-2 infection, as well as side effects of the BNT162 vaccine, were compared between 97 patients with NS and 97 age- and sex-matched healthy subjects.

**Results:**

No statistically significant differences were found in the severity or duration of COVID-19 between NS patients and controls. NS patients exhibited a higher rate of rhinorrhea during SARS-CoV-2 infection (69.2% vs. 46%, *p* < 0.05), whereas anosmia was more common among controls (1.5% vs. 12.7%, *p* < 0.05). No statistically significant differences in side effects from the BNT162 vaccine were observed between groups. 95% confidence intervals were calculated for key outcomes to improve comparability.

**Conclusion:**

Children and adolescents with Noonan Syndrome generally experience a mild course of COVID-19 and show no evidence of increased vaccine-related risk. Despite the small sample size and the rarity of severe events, these findings yield valuable insights for patients living with this rare disease.

## 1 Introduction

Coronavirus disease 2019 (COVID-19), caused by the severe acute respiratory syndrome coronavirus 2 (SARS-CoV-2), was declared a pandemic by the World Health Organization (WHO) between March 2020 and May 2023 (1). The Italian National Institute of Health (ISS) reports 4,884,844 COVID-19 cases in individuals under 19 years, with a 13.3% hospitalization rate, higher in younger age groups, and pediatric mortality below 0.5% (2, 3). Most children and adolescents with SARS-CoV-2 infection are asymptomatic or present with mild symptoms, though clinical manifestations vary widely (4). Common symptoms include fever, cough, vomiting, diarrhea, fatigue, nasal congestion, anosmia, and ageusia. Hospitalized patients may develop pneumonia as well as neurological and cardiac complications (5, 6). Late manifestations such as Long COVID and Pediatric Multisystem Inflammatory Syndrome (MIS-C) have also been observed (5, 7–8). Endocrine and metabolic disorders are known to increase the risk of severe outcomes in COVID-19, reinforcing the importance of monitoring patients with chronic conditions during infection (9, 10).

**Figure 1:**
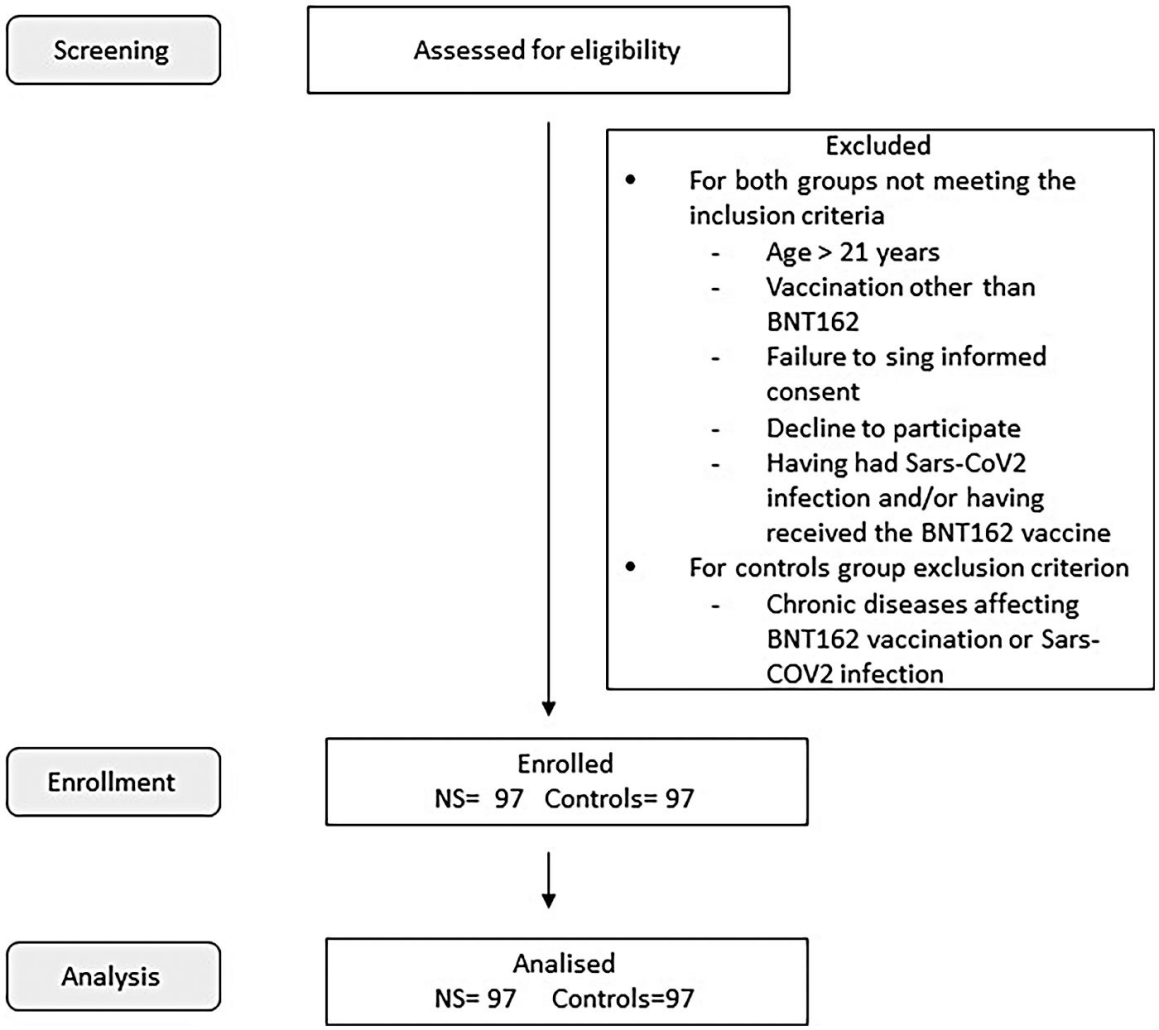
Simple elegibility flow diagram.

The BNT162 (Comirnaty) vaccine has been approved by the WHO for use starting at 6 months of age (11). In Italy, the monovalent formulation targeting the Omicron variant was authorized in September 2023 (12). Efficacy in children aged 5–11 years appears slightly lower than in older children, but studies have demonstrated effectiveness in preventing infection, and especially hospitalization (12, 13). For children under 5 years of age, a Singapore study showed an efficacy of 63.3% against and 74.6% against reinfections (14). The vaccine is associated with rare side effects, such as mild, dose-dependent local or systemic reactions, which resolve within a few days (11, 15, 16). Serious adverse events, including myocarditis and pericarditis, are rare (15, 17). Notably, the risk of cardiac complications from SARS-CoV-2 infection is significantly higher than that from vaccination. Vaccination is still considered the safest method to prevent cardiac complications (15, 17, 18).

Noonan syndrome (NS) is a rare, highly variable, multisystemic disorder, primarily inherited in an autosomal-dominant manner, caused by germline mutations in genes encoding proteins involved in the RAS-MAPK signaling pathway. This key pathway regulates cell proliferation, differentiation, survival, and metabolism (19). Its estimated incidence is 1 in 1,000–2,500 live births (19, 20). NS is characterized by postnatal growth restriction and distinctive facial dysmorphisms, congenital heart defects (CHD), hypertrophic cardiomyopathy, skeletal abnormalities, and webbed neck. Other features include hemorrhagic diathesis, ectodermal anomalies, lymphatic dysplasias, cryptorchidism, and cognitive deficits. NS shows significant phenotypic variability, which can be partly explained by the underlying molecular alterations (19, 21). Molecular genetic testing can confirm approximately 70% of clinically suspected cases (22).

Patients with NS often exhibit immune abnormalities and a higher prevalence of autoimmune diseases such as systemic lupus erythematosus, autoimmune thyroiditis, celiac disease, and autoimmune hepatitis. Additionally, some individuals present with hypogammaglobulinemia or impaired antibody responses, leading to increased susceptibility to infections (23–25). These findings suggest a potential immune dysregulation that may affect both the course of infection and the vaccine response.

The BNT162b2 vaccine has demonstrated variable immune responses in immunocompromised individuals. For instance, patients with common variable immunodeficiency exhibited a measurable antibody response to the vaccine, but at lower titers compared to healthy controls. Similarly, adolescents with autoimmune inflammatory diseases showed impaired *in vitro* neutralization capacity following vaccination. These findings suggest that individuals with immune dysfunction, such as those with NS, may have a diminished ability to mount a robust immune response to the BNT162b2 vaccine (26, 27).

There is no specific treatment for NS management that is tailored to individual symptoms (20). Recombinant human growth hormone (GH) is approved for NS-associated short stature (19). There are currently no published data on the course of SARS-CoV-2 infection or the effects of vaccines in children and adolescents with NS. Therefore, this study aimed to evaluate the incidence, symptoms, and severity of COVID-19, as well as the side effects of the BNT162 vaccine, in a national multicenter cohort of pediatric and adolescent NS patients compared with matched healthy controls.

## 2 Materials and methods

### 2.1 Study design and setting

This multicenter, retrospective-prospective observational study was conducted across ten Italian pediatric centers between March 2020 and December 2022. The study protocol was approved by the Ethics Committee of the coordinating center (Prog. 3877.CESC) and conducted in accordance with the Declaration of Helsinki and Italian privacy regulations. Written informed consent or parental permission was obtained for all participants as appropriate for age; assent was obtained when applicable. The study adhered to the COVID-19 clinical trial assessment procedures issued by the Italian Medicines Agency (AIFA) in force during data collection (28).

### 2.2 Study population

We enrolled 97 children and adolescents with NS and 97 healthy controls matched 1:1 by age and sex. Inclusion criteria for both groups were: age < 21 years; confirmed SARS-CoV-2 infection between March 2020 and December 2022, and/or receipt of BNT162b2 vaccination during the study period.

Exclusion criteria for controls included chronic diseases and ongoing treatments that could potentially affect vaccine response or the course of SARS-CoV-2 infection (e.g., chemotherapy, active malignancy, primary immunodeficiency). malignancy, primary immunodeficiency). Controls were recruited from the same centers and catchment areas as the NS participants (i.e., outpatient clinics and community referrals) to minimize selection bias.

A simple flow diagram (screened → eligible → enrolled  → analyzed) has been included as Supplementary Figure S1 to illustrate participant disposition.

### 2.3 Data collection

COVID-19 was confirmed by RT-PCR or rapid antigen testing on nasopharyngeal swabs, as per national standards.

Clinical information was obtained from medical records; missing items were completed via standardized telephone interviews with caregivers.

To evaluate potential recall bias from telephone interviews, we recorded the interval (in months) between the event and the interview and performed sensitivity analyses stratified by recall window (≤3, 4–6, and >6 months).

Data collected included age at onset, symptoms, duration, treatments, complications, and hospital admissions. Laboratory results were recorded when available**.**

### 2.4 Outcomes

The primary outcomes were clinical severity and duration of COVID-19, as well as adverse events following BNT162b2 vaccination.

COVID-19 severity was categorized as follows (29):

1. Asymptomatic;
2. Paucisymptomatic (dry cough, malaise, headache, low-grade fever, tiredness);
3. Mild (uncomplicated upper respiratory or GI symptoms, with/without fever);
4. Severe (pneumonia, hypoxia, dyspnea, tachypnea, severe dehydration);
5. Critical (severe pneumonia, ARDS, septic shock, MIS-C, or organ dysfunction requiring intensive care).

Duration was defined as the interval between the first positive and the first negative swab.

Vaccine adverse events were classified according to product characteristics (local: pain, redness, swelling; systemic: fever, fatigue, headache, myalgia, chills, nausea; serious: myocarditis/pericarditis, anaphylaxis) (30, 31).

The safety surveillance window for adverse events was 0–30 days post-dose; events outside this interval were recorded if reported by families but analyzed descriptively.

### 2.5 Baseline covariates and subgroups

For NS participants, we collected genotype (e.g., PTPN11, SOS1, RAF1, RIT1, KRAS, BRAF, LZTR1, others), cardiac phenotype (type of CHD and surgical history), endocrine and immunologic comorbidities, and GH therapy status.

Prespecified subgroups included genotype (PTPN11 vs. other), CHD (yes/no), GH therapy (yes/no), and the presence of immune/endocrine comorbidities; given the small cell counts, subgroup analyses were considered exploratory. Baseline immune parameters (immunoglobulin levels, autoimmune comorbidities, history of recurrent infections, and use of immunosuppressive medications) were collected when available. Thrombosis and bleeding events were systematically screened during data collection through a review of medical charts. No specific laboratory assays for thrombotic markers (e.g., D-dimer, fibrinogen) or cardiac injury markers (e.g., troponin, CK-MB) were systematically performed; however, these were extracted when available from clinical records.

### 2.6 Calendar periods (variant waves) and exposure sequence

Because symptom profiles and testing strategies evolved over time, analyses were stratified by calendar periods that approximated the predominant variants (Pre-Delta, Delta, Omicron) based on national surveillance timelines. We also described the sequence of infection and vaccination (infection before vs. after vaccination) and the number of doses received, when available.

### 2.7 Data management and availability

Data were managed using REDCap electronic tools (32). The translated CRF is available as supplementary material.

### 2.8 Statistical analysis

Statistical analysis was performed using IBM SPSS version 26 (Inc., Chicago, IL, USA). Continuous variables are reported as mean ± SD; categorical variables as frequencies and percentages. Group comparisons were performed using the Mann–Whitney *U* test for continuous variables. The category variables were compared using the Chi-square and Fisher's tests. Exact test for categorical variables. Statistical significance was set at *p* < 0.05 (two-tailed).

We report 95% confidence intervals (95% CI) for key binary outcomes (e.g., hospitalization, severe/critical disease, any adverse event) and for selected differences in proportions.

Sensitivity analyses included stratification by recall window and calendar period; subgroup results are presented with explicit sample sizes and should be interpreted cautiously due to the limited power. No multiplicity adjustment was applied, given the exploratory intent; *p*-values are descriptive.

## 3 Results

### 3.1 Study population

A total of 97 NS patients (M/F = 51/46; mean age 11.98 ± 5.34 years) and 97 healthy controls (M/F = 55/42; mean age 12.14 ± 5.19 years) were included. Among NS patients, 35 (36%) had SARS-CoV-2 infection, 32 (33%) received BNT162 vaccination, and 30 (31%) had both during the study period. Among controls, 28 had SARS-CoV-2 infection, 34 received BNT162 vaccination, and 35 had both.

### 3.2 Genetic and clinical features of NS patients

The genotype distribution included PTPN11 (65; 67%), SOS1 (7; 7.2%), RAF1 (2; 2%), RIT1 (3; 3.1%), KRAS (6; 6.2%), BRAF (1; 1%), LZTR1 (6; 6.2%), and other variants (7; 7.2%).

Seventy-two NS patients (74.2%) had congenital heart disease (CHD), most commonly pulmonary valve stenosis (44; 45.4%), atrial/ventricular septal defects (27; 27.9%), and hypertrophic cardiomyopathy (11; 11.3%).

Subgroup sample sizes for genotype and CHD are reported with each analysis below to emphasize limited power for rare strata.

Table 1 details the comorbidities reported by NS patients, including their respective percentages (Table 1).

**Table 1:** Comorbidities reported by Noonan syndrome (NS) patients.

Associated condition	Number of patient (%)
Hearth condition	72 (74.2%)
Coagulation abnormalities	10 (10.3%)
Psychomotor developmental anomalies	51 (52.6%)
Visual system anomalies	48 (49.5%)
Genitourinary anomalies	36 (37.1%)
Skeletal anomalies	41 (42.3%)
Hearing loss	4 (4.1%)
DNET	3 (3.1%)
Immune deficiency	3 (3.1%)
Autoimmune thyroiditis	3 (3.1%)
Chiari malformation	3 (3.1%)
Headache	3 (3.1%)
Asthma	2 (2.1%)
Hypothyroidism	2 (2.1%)
Hypoplasia of the cerebellar vermis	2 (2.1%)
Malocclusion	2 (2.1%)
Gallstones	2 (2.1%)
Mega cisterna magna	1 (1%)
Bilateral temporo-polar cysts	1 (1%)
Dandy-Walker malformation	1 (1%)
Optic nerve atrophy	1 (1%)
Syringomyelia	1 (1%)
Hyposmia	1 (1%)
Myelomonocytic leukemia	1 (1%)
Phenylketonuria	1 (1%)
Hypergonadotropic hypogonadism	1 (1%)
Seminiferous tubule fibrosis	1 (1%)
Mega cisterna magna	1 (1%)
Phenylketonuria	1 (1%)
Hypergonadotropic hypogonadism	1 (1%)
Seminiferous tubule fibrosis	1 (1%)
Bilateral temporo-polar cysts	1 (1%)
Splenomegaly	1 (1%)
Hepatic steatosis	1 (1%)
Congenital aganglionic megacolon	1 (1%)
APLV	1 (1%)
Food allergies	1 (1%)
Celiac disease	1 (1%)
Cystic fibrosis	1 (1%)
Thyroid cyst	1 (1%)
Hyposmia	1 (1%)
Myelomonocytic leukemia	1 (1%)

### 3.3 COVID-19 clinical course

Infection rates were similar between NS (65/97; 67%) and controls (63/97; 65%). Table 2 presents a comparative analysis between NS patients and controls regarding the primary symptoms of COVID-19 (Table 2).

**Table 2:** Symptoms reported during COVID-19, Pearson's and Fisher's test.

Symptoms	NS (%)	Controls (%)	Pearson's Chi-square	Fisher's Test
	IC 95%	IC 95%		
Fever	41 (63.1%)	42 (66.7%)	.671	.405
	[0.32, 0.52]	[0.33, 0.53]		
Cough	28 (43.1%)	28 (44.4%)	.876	.509
	[0.20, 0.38]	[0.20, 0.38]		
Rhinorrhea	45 (69.2%)	29 (46%)	.008*	.012
	[0.36, 0.56]	[0.21, 0.39]		
Pharyngodynia	22 (33.8%)	18 (28.6%)	.520	.326
	[0.14, 0.31]	[0.11, 0.26]		
Anosmia	1 (1.5%)	8 (12.7%)	.009*	.014
	[0.00, 0.03]	[0.03, 0.14]		
Ageusia	2 (3.1%)	6 (9.5%)	.124	.127
	[0.00, 0.05]	[0.01, 0.11]		
Headache	11 (16.9%)	16 (25.4%)	.239	.169
	[0.05, 0.18]	[0.09, 0.24]		
Arthralgias	7 (10.8%)	11 (17.5%)	.275	.202
	[0.02, 0.12]	[0.05, 0.18]		
Diarrhea	6 (9.2%)	4 (6.3%)	.542	.392
	[0.01, 0.11]	[0.00, 0.08]		
Vomiting	7 (10.8%)	2 (3.2%)	.084	.090
	[0.02, 0.12]	[0.00, 0.05]		
Vasculitis	0 (0%)	0 (0%)		
	0	0		
Pneumonia	1 (1.5%)	0 (0%)	.243	.508
	[0.00, 0.03]	0		
Other symptoms	5 (7.7%)	3 (4.8%)	.541	.407
	[0.01, 0.10]	[0.00, 0.07]		
Complications	1 (1.5%)	3 (4.8%)	.278	.292
	[0.00, 0.03]	[0.00, 0.07]		
Drug use	35 (53.8%)	39 (61.9%)	.376	.229
	[0.42, 0.66]	[0.50, 0.74]		

* *P* < 0.05.

Age at diagnosis of SARS-CoV-2 infection (10.80 ± 5.22 vs. 11.03 ± 5.15 years), duration of symptoms (4.54 ± 3.19 vs. 4.48 ± 2.92 days), and time to negative test (10.37 ± 3.99 vs. 10.97 ± 5.12 days) were not statistically significant between the two groups. The most frequently reported symptoms were rhinorrhea, fever, cough, pharyngodynia, and headache.

Anosmia was less common in NS (1.5%) than in controls (12.7%) (*p* < 0.05), whereas rhinorrhea was more frequent in NS (69.2% vs. 46%) (*p* < 0.05).

For these contrasts we provide 95% CI for the difference in proportions in Table 2. Nearly all cases were managed at home with mild or moderate.

One NS patient (infected at 1 year of age; ICU at 4 years) required intensive care due to hyponatremic dehydration; two additional NS patients required standard hospital admission (infected at 7 and 11 years, respectively). Among controls, one 14-year-old female was hospitalized for menometrorrhagia with severe anemia. Medication was used in 35 NS (53.8%) and 39 controls (61.9%); paracetamol was the most frequently used drug. One NS patient received oxygen (2 L/min for 2 days), and one received monoclonal antibodies; no patient required changes to their baseline therapies, and disease control remained stable both pre- and post-infection.

### 3.4 Exploratory subgroup analyses

Among NS patients, no statistically significant differences were found in the course of SARS-CoV-2 infection in terms of the total number and types of symptoms reported, duration of symptoms in days (4.41 ± 2.85 in patients with CHD VS 4.88 ± 4.01 in patients without CHD), time to negative test result (10.6 ± 4.33 in patients with CHD VS 9.76 ± 2.91 in patients without CHD), and severity of the infection (mild symptoms in 75% of CHD patients VS 78.6% of patients without CHD) when comparing patients with congenital heart disease to those without, or when comparing patients receiving GH therapy to those not receiving therapy.

However, a higher incidence of vomiting was observed during SARS-CoV-2 infection in patients with NS and genitourinary anomalies (26.3%) compared to those without (4.3%) (*p* < 0.05). When comparing NS patients based on other possible organic or psychomotor anomalies, no significant differences were found between those with and without anomalies. Given small counts, this finding warrants cautious interpretation and external validation.

### 3.5 Calendar periods and exposure sequence

Descriptively, the majority of infections occurred during late 2021–mid 2022 (Delta/Omicron periods). Severity and time-to-negativity appeared similar across calendar periods, although variant attribution was not available at the individual level.

Where information was available, we described whether infection preceded vaccination or occurred after partial/complete vaccination. No consistent pattern in symptom severity was observed; nonetheless, the numbers were small.

### 3.6 Data analysis of the BNT162 vaccine

BNT162 vaccination was administered to 62 NS patients (63.9%) and 69 controls (71.1%). The mean age at vaccination showed no statistically significant differences between the two groups (12.90 ± 4.14 vs. 12.74 ± 3.97 years). Regarding those who had contracted COVID-19 before receiving the vaccination, 6 NS patients and 10 subjects in the control group had a prior history of infection. The most common adverse events in both groups were injection-site pain, redness, fever (>37.5 °C), and fatigue; no serious adverse events were reported. As shown in Table 3, no statistically significant differences were found in the frequency of side effects reported between the two groups, nor in the number of side effects reported per individual (Table 3).

**Table 3:** Sides effects of BNT162 vaccine, Pearson's and Fishers test.

Sides effects	NS (%)	Control (%)	Pearson's Chi-square	Fisher's test
	IC 95%	IC 95%		
Injection site pain	41 (66.1%)	36 (52.2%)	.104	.074
	[0.32, 0.52]	[0.28, 0.47]		
Redness at the injection site	11 (17.7%)	7 (10.1%)	.207	.157
	[0.05, 0.18]	[0.02, 0.12]		
Itching at the injection site	3 (4.8%)	4 (5.8%)	.807	.560
	[0.00, 0.07]	[0.00, 0.08]		
Swelling at the injection site	6 (9.7%)	4 (5.8%)	.403	.306
	[0.01, 0.11]	[0.00, 0.08]		
Swelling of the lymph nodes	1 (1.6%)	4 (5.8%)	.195	.218
	[0.00, 0.03]	[0.00, 0.08]		
Temperature > 37.5 °C	9 (14.5%)	9 (13.0%)	.807	.503
	[0.04, 0.15]	[0.04, 0.15]		
Headache	6 (9.7%)	7 (10.1%)	.929	.582
	[0.01, 0.11]	[0.02, 0.12]		
Arthralgias	5 (8.1%)	5 (7.2%)	.860	.558
	[0.01, 0.10]	[0.01, 0.10]		
Abdominal pain	0 (0%)	0 (0%)		
	0	0		
Diarrhea	0 (0%)	0 (0%)		
	0	0		
Vomiting	0 (0%)	1 (1.4%)	.256	.527
	0	[0.00, 0.03]		
Insomnia	0 (0%)	0 (0%)		
	0	0		
Fatigue	10 (16.1%)	9 (13.0%)	.617	.400
	[0.04, 0.16]	[0.04, 0.15]		
Myocarditis/Pericarditis	0 (0%)	0 (0%)		
	0	0		
Herpes zoster	0 (0%)	0 (0%)		
	0	0		
Anaphylactic shock	0 (0%)	0 (0%)		
	0	0		
Other symptoms	0 (0%)	2 (2.9%)	.107	.276
	0	[0.00, 0.05]		
Drug use	14 (22.6%)	11 (15.9%)	.399	.268
	[0.12, 0.33]	[0.08, 0.26]		

Cardiac enzymes/ECG/echocardiography were performed only if clinically indicated; as systematic screening was not undertaken, subclinical myocarditis cannot be entirely excluded.

Prior infection status (yes/no) did not significantly alter the frequency or pattern of post-vaccination adverse events in either group; however, the strata were small (NS prior infection, *n* = 6; controls, *n* = 10).

Following the onset of side effects, 14 NS patients and 11 healthy controls required medication, primarily paracetamol.

## 4 Discussion

### 4.1 Main findings

This is the first study to investigate the clinical course and severity of SARS-CoV-2 infection, as well as the side effects of the BNT162 vaccine, in children and adolescents with Noonan Syndrome (NS). Our findings showed no statistically significant differences in infection severity, symptom duration, or viral clearance between NS patients and healthy controls.

Patients with NS often present with various comorbidities, particularly congenital heart defects (CHD), which may represent risk factors for more severe COVID-19 (19, 32).

Few studies have evaluated the severity of SARS-CoV-2 infection in patients with genetic syndromes and/or CHD. A study conducted by the American Heart Association suggests that patients with CHD and genetic syndromes may be at higher risk for severe SARS-CoV-2 infection and adverse outcomes due to comorbidities, potential alterations in immune response, and overall health status. In contrast, patients with well-compensated CHD alone do not exhibit worse infection severity (33). Other reports indicated a 50%–75% higher risk of hospitalization and mortality among patients with CHD than among controls, though these associations were influenced by comorbidities such as pulmonary hypertension rather than cardiac complexity (34, 35).

The correlation between COVID-19 and the presence of genetic syndromes and CHD in the pediatric population is less explored. Some studies have found that children and adolescents with CHD are at higher risk for complications and longer hospital stays during COVID-19 (36, 37). However, the risk of mortality and hospitalization remains lower than that of the general population, even in individuals with severe CHD (37, 38).

To our knowledge, there are no specific studies on CHD in patients with NS and COVID-19. However, the literature reports the case of a 5-year-old child who suddenly died due to SARS-CoV-2 infection. The subsequent autopsy revealed the presence of NS, with coronary artery malformation and B-cell lymphoblastic leukemia (BCP-ALL). This article suggests that SARS-CoV-2 infection may have been the triggering factor for sudden cardiac death due to increased cardiac load (39).

In our population, despite 74.2% of patients with NS having CHD, no statistical differences in infection severity were observed. This finding aligns with literature suggesting that pediatric patients with stable CHD generally experience mild COVID-19 courses. The mild course observed in our cohort may reflect several factors:

1. Children and adolescents generally experience less severe SARS-CoV-2 infection;
2. Most cardiac conditions in our NS population were mild or surgically corrected with adequate hemodynamic compensation;
3. All participants had stable underlying disease control during the pandemic period.

A previous study by Lewis et al. describes how patients with CHD and a diagnosis of Down syndrome or DiGeorge syndrome were at higher risk for complications during COVID-19 infection (33). This supports earlier reports that RASopathy patients rarely show clinically significant immunodeficiency (40). However, the absence of significant differences in our cohort suggests that most NS patients, particularly those with compensated or surgically corrected CHD, did not experience worse outcomes. Nevertheless, given the relatively small sample and the rarity of severe events, our results should not be interpreted as definitive evidence of equivalent risk but rather as reassurance that the majority of NS patients do not appear to be at high risk for severe infection.

Our study found a higher incidence of anosmia in healthy controls compared to non-smoking patients (NS) (12.7% vs. 1.5%, *p* < 0.05). The etiopathogenesis of anosmia associated with COVID-19 is not fully understood, and multiple mechanisms have been proposed. Among these, local inflammation of the olfactory epithelium following viral infection, with increased TNF-α production and IL-6 levels as regulators of apoptosis in olfactory epithelial cells, seems particularly important (41). Since the dysfunction of the RAS-MAPK pathway, typical of RASopathies like NS, also involves mechanisms of inflammatory response and cell proliferation, this may explain the lower incidence of anosmia in NS patients (19). The proposed biological mechanism—impaired inflammatory signaling in the RAS-MAPK pathway—remains speculative. Further studies assessing cytokine profiles and mucosal immune responses in NS could clarify whether altered inflammation modulates olfactory outcomes.

Conversely, rhinorrhea was more frequent among NS patients (69.2% vs. 46%, *p* < 0.05). This may reflect anatomical differences such as adenoid hypertrophy or chronic nasal obstruction, which have been reported in NS children (42–45). It could be speculated that nasal obstruction due to adenoid hypertrophy might predispose to a higher rate of rhinorrhea during viral infections such as COVID-19. Adenoid hypertrophy in children is linked to chronic inflammation and immune dysregulation, with altered cytokine profiles and lymphocyte populations contributing to tissue enlargement. This biological mechanism supports the hypothesis that nasal obstruction due to adenoid hypertrophy may predispose children to increased rhinorrhea during viral infections, including COVID-19. However, current evidence remains insufficient to confirm this definitively, and further research is needed (46).

### 4.2 Vaccine safety

Our data confirm the favorable safety profile of the BNT162 vaccine in NS patients. The frequency and type of adverse events were comparable between groups, with the majority being mild and self-limiting (e.g., injection-site pain, fatigue, fever). No serious adverse events (e.g., myocarditis or pericarditis) were reported. Our findings are consistent with those previously reported in the literature, where the most common symptoms reported post-vaccination in children and adolescents include pain at the injection site, fatigue, and headache (15, 17, 47). Moreover, no significant differences were observed in the number of vaccinations between patients with NS and healthy controls. This suggests that patients with NS did not view themselves as being at a higher risk for severe SARS-CoV-2 infection and adhered to the AIFA guidelines for vaccination.

The immune system abnormalities associated with NS include both immunodeficiency and immune dysregulation. The recent case involving a 14-year-old girl with NS, caused by a variant in the MAPK1 gene, showed signs of recurrent infections, hypogammaglobulinemia, and impaired antibody responses, which indicate a combined immunodeficiency. This immune dysfunction may stem from the dysregulation of the Ras-ERK and PI3K-AKT-mTOR signaling pathways (23–25). Additionally, autoimmune conditions such as celiac disease and systemic lupus erythematosus have been observed in patients with NS, highlighting the need for a comprehensive immunological evaluation, especially for those experiencing recurrent infections or autoimmune symptoms.

In our study, none of our patients exhibited mutations in MAPK1, and no significant differences were noted in patients with NS who also had associated autoimmune conditions. NS is associated with both immune dysregulation and autoimmunity. While these features raise theoretical concerns about vaccine reactogenicity or efficacy, our findings support that BNT162 remains safe for this population. Future research should include immunogenicity end-points (antibody titers and cellular responses) to determine whether immune activation differs by genotype or clinical phenotype.

### 4.3 Strengths and limitations

The strengths of this study include its multicenter design, the largest national NS cohort reported to date for a rare disease, and a matched control group. The study's main limitations include its retrospective nature and the potential for recall bias from telephone interviews. However, it is essential to emphasize that the global health emergency caused by the SARS-CoV-2 coronavirus made the experience of infection and/or vaccination an unforgettable event for many, thereby mitigating the risk of recall distortions. To address this issue, we performed a sensitivity analysis by time window of recall and acknowledged that misclassification of mild symptoms could bias results toward the null. Another limitation of our study is the lack of information on the specific SARS-CoV-2 variants in the analyzed cases. Although we intended to include this aspect, the molecular and/or rapid diagnostic tests routinely used for infection detection did not provide variant-level data. Considering that most infections occurred between December 2021 and July 2022, it is reasonable to assume that the predominant variants were Delta and Omicron. Since the study period encompassed both variant waves, potential variant-specific effects cannot be ruled out, as genotyping data were unavailable.

Systematic follow-up for MIS-C or long COVID was not conducted, and only data from the acute phase were collected. This limitation was acknowledged and considered when interpreting the study's safety findings.

### 4.4 Clinical implications

Our results suggest that children and adolescents with Noonan Syndrome are not at increased risk of severe COVID-19 or vaccine-related adverse events compared with healthy peers. These data support the safety and efficacy of COVID-19 vaccination in this population and may help inform clinical practice and vaccination policies. However, given the rarity of NS and limited statistical power for uncommon outcomes, ongoing surveillance and collaborative international registries are recommended**.**

## 5 Conclusions

In this multicenter study, we did not observe greater severity of SARS-CoV-2 infection in children and adolescents with Noonan Syndrome compared with controls. The overall course of COVID-19 was mild and uncomplicated in most participants. One unvaccinated 2-year-old required intensive care for hyponatremic dehydration. This isolated case underscores the importance of timely vaccination in eligible children. The safety profile of the BNT162 vaccine was confirmed in NS patients. These findings support COVID-19 vaccination as a safe preventive strategy in this population.

Further prospective studies should include immunological follow-up to assess the durability of vaccine responses and potential genotype-specific effects.

## Data Availability

The datasets presented in this article are not readily available because this option was not included in the informed consent form signed by the parents, and the Ethics Committee did not authorize the public dissemination of individual-level data, even in anonymized form. In accordance with national privacy regulations and GDPR requirements, the dataset can be made available only upon a reasonable request to the corresponding author, as explicitly approved by the Ethics Committee.
