# Supplementary material for: Incidence and severity of SARS-CoV-2 infection and vaccine BNT162 side effects in children and adolescents with Noonan Syndrome: a national multicentric study
Source: Front Pediatr. 2026 Jan 12;13:1658340. doi: 10.3389/fped.2025.1658340 (PMC12832667; doi:10.3389/fped.2025.1658340)
Supplement: Supplementary file 2 [file Supplementaryfile2.docx]

**Form 2: Follow-Up Controls - Clinical Data Collection**

| Record ID |  |
| --- | --- |
| Name of the center completing the form |  |
| Informed Consent | Date of informed consent collection  Upload informed consent |
| Personal Information | Date of birth  Age |
| Ethnicity | - Caucasian - Non-Caucasian - Unknown |
| Gender | - Female - Male - Not Specified |
| Underlying Conditions | Yes / No  If yes, specify |

**COVID-19 INFECTION**

| Infection with COVID-19 | Yes / No |
| --- | --- |
| Date of COVID-19 diagnosis |  |
| Age at diagnosis |  |
| Variant type (if available) |  |
| Signs and Symptoms of Infection | - Fever - Cough - Rhinorrhea - Pharyngodynia - Anosmia - Ageusia - Headache - Arthralgia - Abdominal pain - Diarrhea - Vomiting - Vasculitis (Kawasaki/MIS-C) - Pneumonia - Other (specify) |
| Complications | Yes / No  Type of complications |
| Overall severity (per protocol) | Mild / Moderate / Severe |
| Symptom duration |  |
| Management of infection | - At home - Hospitalized (standard care) - Hospitalized (intensive care) |
| Need for medications | Yes / No  If yes, specify (dosage and duration) |
| Need to modify baseline therapy | Yes / No  If yes, how and for how long |
| Time from diagnosis to negative swab (in days) |  |

**COVID-19 VACCINATION**

| Vaccinated against COVID-19 | Yes / No |
| --- | --- |
| Date of vaccination |  |
| Age at vaccination |  |
| Type of vaccine |  |
| Had COVID-19 before vaccination | Yes / No |
| Side effects | - Pain at injection site - Redness at injection site - Itching at injection site - Swelling at injection site - Lymph node enlargement - Temperature &gt; 37.5°C - Headache - Arthralgia - Abdominal pain - Diarrhea - Vomiting - Insomnia - Fatigue - Myocarditis/Pericarditis - Lymphadenopathy - Herpes zoster infection - Anaphylactic shock - Other |
| Medications for side effects | Yes / No  Specify (dosage and duration) |
